# Historical Differences in School Term Length and Measured Blood Pressure: Contributions to Persistent Racial Disparities among US-Born Adults

**DOI:** 10.1371/journal.pone.0129673

**Published:** 2015-06-15

**Authors:** Sze Yan Liu, Jennifer J. Manly, Beatrix D. Capistrant, M. Maria Glymour

**Affiliations:** 1 Harvard Center for Population and Development Studies, Harvard School of Public Health, Cambridge, Massachusetts, United States of America; 2 Gertrude H. Sergievsky Center, Taub Institute for Research on Alzheimer’s Disease and The Aging Brain, and Department of Neurology, Columbia University Medical Center, New York, New York, United States of America; 3 Division of Epidemiology and Community Health, School of Public Health, University of Minnesota, Minneapolis, Minnesota, United States of America; 4 Department of Epidemiology & Biostatistics, University of California San Francisco School of Medicine, San Francisco, California, United States of America; School of Public Health, Zhejiang University, CHINA

## Abstract

**Introduction:**

Legally mandated segregation policies dictated significant differences in the educational experiences of black and white Americans through the first half of the 20^th^ century, with markedly lower quality in schools attended by black children. We determined whether school term length, a common marker of school quality, was associated with blood pressure and hypertension among a cohort of older Americans who attended school during the *de jure* segregation era.

**Methods:**

National Health and Nutrition Examination Survey I and II data were linked to state level historical information on school term length. We used race and gender-stratified linear regression models adjusted for age, state and year of birth to estimate effects of term length on systolic and diastolic blood pressure (SBP and DBP) and hypertension for US-born adults. We also tested whether correcting years of schooling for term length differences attenuated estimated racial disparities.

**Results:**

Among black women, 10% longer school term was associated with lower SBP, DBP and hypertension prevalence (2.1 mmHg, 1.0 mmHg, and 5.0 percentage points respectively). Associations for whites and for black men were not statistically significant. Adjustment for education incorporating corrections for differences in school term length slightly attenuated estimated racial disparities.

**Conclusions:**

Longer school term length predicted better BP outcomes among black women, but not black men or whites.

## Introduction

Hypertension and blood pressure are strong predictors of cardiovascular disease, the leading cause of death in the world and in the US.[1–3] Black Americans have higher cardiovascular disease rates and mortality rates than whites Americans,[3,4] and hypertension is considered a key contributor to these racial disparities.[5,6] Previous research has suggested educational, rather than genetic, differences drive racial disparities in hypertension.[7] However, while education contributes to these racial inequalities, racial differences in hypertension persist even after controlling for educational attainment.[8]

A large body of research indicates education is strongly associated with lower blood pressure and hypertension.[9–11] The difference in hypertension prevalence between individuals with post- secondary degrees to those with HS degrees [12] is similar in magnitude to reductions in hypertension achieved by successful community-based risk behavior interventions.[13,14] Given our knowledge that racial and ethnic patterns in adult health may reflect earlier macrosocial inequities [15–18], it is important to understand how historical educational policies may explain the persistent racial disparity in hypertension.

While much previous research on education and health has examined education as a degree credential or years of schooling completed, an emerging body of work addresses how educational quality and other structural components of education may affect health.[19–21] Health returns for individuals with the same educational attainment may vary because of qualitative dimensions in the educational experience such as differences in school term length. Previous research on education and hypertension using years of schooling does not account for differences in school term length across time, geographic region and race. This may be particularly problematic in understanding the role of education in racial health disparities because many older black adults in the US lived through the *de jure* segregation period.

Adjusting for years of schooling, without correction for any indicators of school quality, may not fully account for racial disparities attributable to differences in educational experiences.

School term length (STL) refers to the number of days schools were legally required to hold classes each year. STL is a state-level school quality indicator reflective of financial resources available to schools within the state and correlated with greater learning opportunities and more material covered in a given school year.[22] Prior to *Brown vs*. *Board of Education* [23], states with *de jure* school segregation usually mandated shorter school term length for black vs. white schools. As a result, school term length differed dramatically for black and white children inmuch of the US in the first half of the 20th century. For example, in 1931, the mandatedminimum school term length for black schools in Alabama was 127 days, compared to 159 days for white schools; a black child in the same grade would therefore average 20% less in-class time than a white child. Because of this, historical racial differences in school term length systematically disadvantaged blacks and adjustment for years of schooling completed does not fully account for this disadvantage.

Furthermore, there may be race- and gender-specific effects of educational quality. Previous research indicates larger educational effects among women compared to men, suggesting there is a resource substitution effect where socially disadvantaged groups are more reliant on various educational attributes to obtain health benefits.[24,25] Socially disadvantaged individuals may be more dependent on education for their health because they have fewer alternatives.[25] Moreover, investigations of a single disadvantaged status in isolation (i.e., examining race but ignoring gender) may obscure important within-group variation.[26] Black females, whose gender and race places them at “multiple jeopardy” [27], may have greater reliance on education for health gains than the other subpopulations.

The purpose of this study was two-fold: 1) to examine the race- and gender-specific associations between school term length and systolic blood pressure (SBP), diastolic blood pressure (DBP) and hypertension in US adults and 2) to evaluate whether incorporating corrections for differences in school term length in the measure of years of schooling leads to a larger attenuation of racial disparities in hypertension when adjusting for “corrected” years of education compared to adjusting for an “uncorrected” measure of years of education. We evaluated whether longer school term lengths in primary school predicts lower SBP and DBP in adulthood, in race and gender-specific subpopulations (i.e. black females, black males, white females and white males). We hypothesized that longer school term lengths in primary school would be associated with lower SBP and DBP in adulthood, especially among socially disadvantaged groups (i.e. black females). We also hypothesized that correcting the years of schooling measure for differences in school term length would lead to a decrease in the estimated effect of race in the association between years of schooling and blood pressure.

## Methods

We used data from the two waves of the National Health and Nutrition Examination Survey (NHANES) which included state of birth—NHANES I, conducted 1971–1975, and NHANES II, conducted 1976–1980. Respondents provided sociodemographic information including birth year, race, education and income. A subset of persons aged 25–74 in both waves received a detailed medical exam. Our sample consisted of black or white individuals born in the US between 1911 and 1945, birth cohorts who attended school during the school segregation period. School term length information was obtained from the Biennial Surveys of Education documents from 1919 to 1957. We merged the state-level school term length to the individual NHANES data using the state of birth and birth year information. For example, an individual who was born in Alabama in 1911 was matched to Alabama school term length data from the years 1919–1925, the time when the individual 8–12 years old and legally required to attend school. The final sample size was 13,954 respondents from all the US states and DC except for Tennessee and Alaska. We conducted sensitivity analyses excluding 2,887 individuals who resided in a stratum without representation from at least one of the four subpopulations (black females, black males, white males and white females) and estimates (results not shown) were similar to the results from the full sample.

Our outcomes were systolic and diastolic blood pressure (SBP and DBP) and hypertension (defined as SBP ≥140 mg/Hg or DBP ≥90 mg/ Hg). A physician measured the BP for participating NHANES adult respondents. A subsample of NHANES participants also had their blood pressure taken two more times by a nurse. To ensure adequate power, we used the physician measured BP. The correlation between the mean values taken by the nurse and the physician was high in the subsample with all three readings (0.86 and 0.83 for SBP and DBP respectively).

We calculated the average school term length at age 8–12 for each individual. Average school term length varied between states and across birth cohorts within the same state. School term length is a meaningful and objective indicator of school quality that has been consistently recorded in a standardized fashion across the US through most of the 20th century STL is uniquely suitable for the type of analyses undertaken here, because few other measures of school quality could be compared across so many states and years. Substantial variability in school term length across geographic areas and time exist because individual states annually determine minimum length of school term; in states with legal racial segregation of schools, term length was routinely specified separately for schools serving white children and those designated for black children.[28] School term length was available on a biennial basis; we used linear interpolation to approximate school term length for the interim years between reports. Similarly, we calculated average annual days of school attendance for each state/year/race birth co hort at ages 8–12. School term length was natural log-transformed to account for “diminishing returns” to additional classroom time suggested in the educational achievement literature.[29]

We also created a “corrected” years of schooling measure, expected years of schooling completed if everyone experiences a 180 day term. We calculated it as the product of years of schooling and average days in the school term for a respondent, divided by 180 days (currently the standard school term length). For example, an individual who reported 12 years of schooling with an average school term length of 155 days would have 10 years of schooling after correction for school term length. Exposure measures were centered at the race-specific or gender-specific sample mean appropriate for the stratified analysis.

We adjusted for sociodemographic variables that are potential confounders [30–32]: age centered at the sample mean (linear and quadratic terms), indicator variables for state of birth and indicator variables for birth year. The state-of-birth indicator variables account for any potential time-invariant state-specific confounders that affected all birth cohorts similarly, and the year-of-birth indicator variables account for birth-cohort specific patterns that affected all states similarly. These state-of-birth and year-of-birth indicator variables account for time-constant differences between states and for nation-wide differences between years of birth. We also included an indicator variable denoting the sample respondents were originally from (NHANES I vs. NHANES II) to account for possible unknown differences in measurement or sample.

We estimated gender- and race-stratified linear regression models to examine the effect of school term length on blood pressure and hypertension. For blood pressure, the results from these models are interpreted as the estimated difference in the outcome (mmHg) associated with a 10% change in average school term length (i.e., β1 times the natural logarithm of 1.10.[33] For hypertension, the results from these linear regression models are interpreted as the *difference in risk* for hypertension associated with a 10% change in average school term length. All analyses were repeated using average days of school attendance instead of school term length.

For the second aim of our paper, we compared regression coefficients for uncorrected years of schooling vs. corrected years of schooling predicting SBP, DBP, and hypertension. Finally, we noted whether there was any attenuation in the racial disparity in SBP, DBP, and hypertension achieved by adjusting for uncorrected versus corrected years of schooling completed. To evaluate whether correction for STL differences increased the attenuation associated with education adjustment, we bootstrapped (500 replications) the difference between the race coefficients from models including the STL uncorrected versus the STL corrected years of education measure. To account for the use of data from both NHANES I and II, as per CDC recommendations, we renumbered the strata so they did not overlap between the surveys. All analyses accounted for the complex survey sampling design but do not incorporate any weights. Analyses were conducted in Stata 11.0. This study was determined by the Harvard School of Public Health Institutional Review Board to be IRB exempt.

## Results

**Fig 1:**
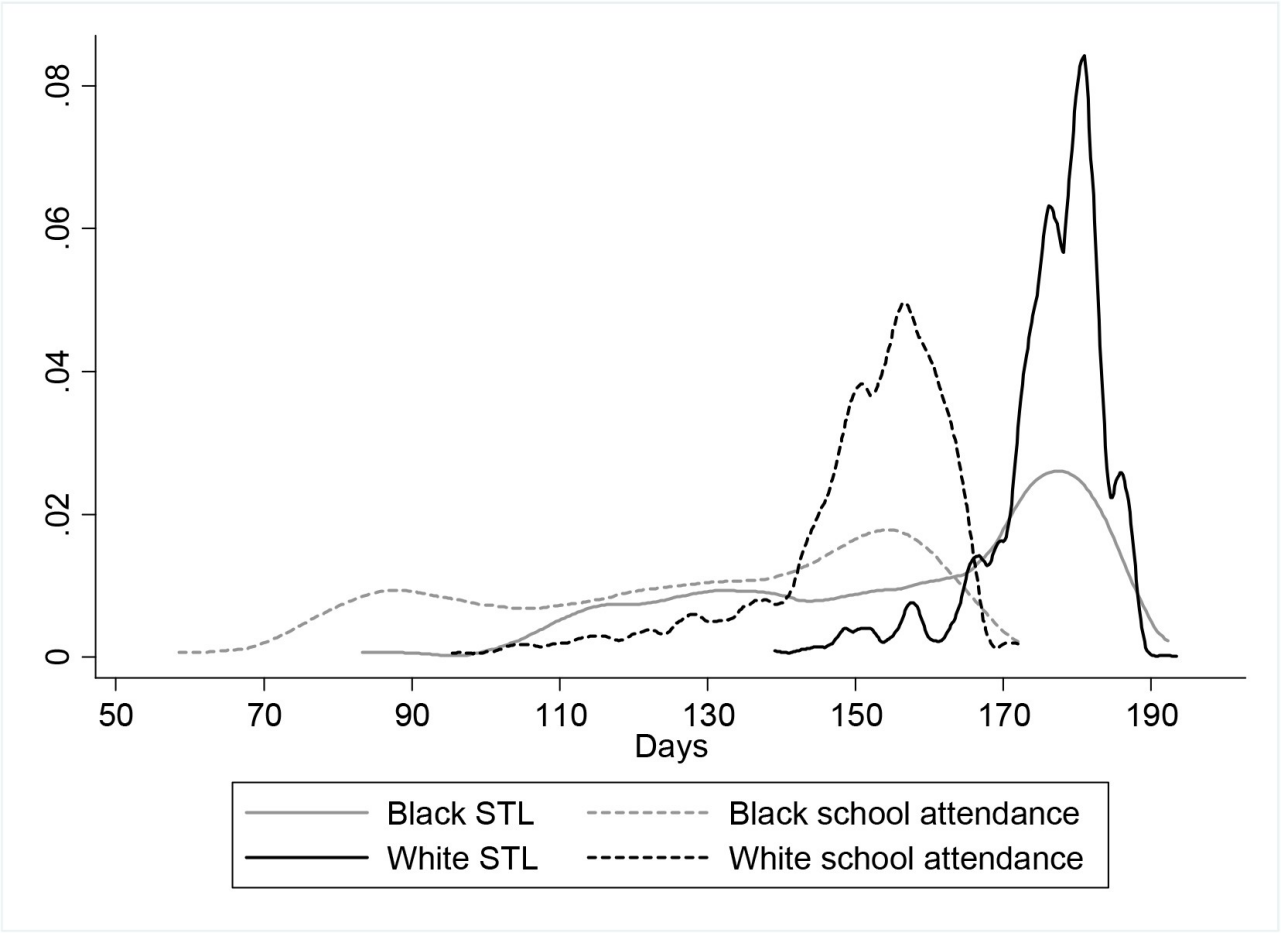
Distribution of average school term length and school attendance at age 8–12 by race, NHANES I & II^e^. Solid gray line indicates average Black school term length; Solid black line indicates average White school term length; Dotted gray line indicates average Black school attendance; Dotted black line indicates average White school attendance.

The distribution of average school term length (Fig 1) differed significantly by race (p-value for Kolmogorov-Smirnov test<0.01), with black respondents experiencing a school term length almost twenty days lower than white respondents (158 vs. 176 days). Over 9 years of primary school, this discrepancy in term length would add up to the equivalent of nearly an entire extra year of school for white children compared to black children. Blacks were also more likely to be from the South, overweight/ obese and hypertensive than whites (Table 1).

**Table 1:** Characteristics of sample members, by sex and race, NHANES I & II.

	Black		White	
	Female	Male	Female	Male
N	1252	704	6919	5079
**Age (range)**	43	47	45	47
	(25–68)	(25–69)	(25–68)	(25–68)
**Birth cohort**				
1911–1915	11	14	13	16
1916–1920	11	17	13	16
1921–1925	10	16	11	14
1926–1930	16	13	14	13
1931–1935	17	11	15	13
1936–1940	17	12	16	13
1941–1945	18	17	18	15
**Years of schooling (%)**				
0–9	26	36	13	17
8–11	32	23	18	16
12 and higher	41	40	69	67
**School term length in days (range)**	158	153	176	175
	(83–192)	(83–191)	(139–194)	(139–194)
**Years of schooling (range)**	10	9	12	12
	(0–17)	(0–17)	(0–17)	(0–17)
**Corrected years of schooling (range)**	9	8	11	12
	(0–17)	(0–17)	(0–18)	(0–18)
**Managerial/Professional (%)**	13	12	26	35
**Systolic BP (range)**	135	138	127	132
	(86–270)	(90–270)	(78–300)	(70–240)
**Diastolic BP (range)**	87	90	81	85
	(25–170)	(50–180)	(35–175)	(40–150)
**Hypertension (%)**	50	57	33	47

The largest differences in blood pressure and probability of hypertension associated with school term length were among black women (Table 2). Among black women, a 10% longer school term length was associated with a 2.1 mmHg (95% CI: -4.1, -0.1) lower SBP, 1.0 mmHg (95% CI: -2.2, -0.1) lower DBP, and 5.0 percentage points (95% CI: -8.4, -1.7) lower hypertension prevalence in the models adjusted for age, state of birth, and birth year (Table 2). No statistically significant associations between school term length and blood pressure or hypertension risk were found among black men, white men or white women. Using average school attendance in lieu of school term length yielded similar estimates and statistical significance.

**Table 2:** Estimated differences and 95% confidence intervals for systolic and diastolic blood pressure and prevalence of hypertension associated with longer school term length and school term attendance, by sex and race, NHANES I & II^a^,^b^.

**Difference in outcome associated with a 10% difference in STL**				
Outcome	Black Female	Black Male	White Female	White Male
Systolic BP(mm/Hg)	-2.1	-1.5	0.4	1.8
	(-4.1, -0.1)	(-4.2, 1.1)	(-1.6, 2.3)	(-0.5, 4.2)
Diastolic BP(mm/Hg)	-1.0	-0.5	1.0	0.5
	(-2.2, 0.1)	(-2.9, 0.7)	(0.1, 1.9)	(-0.9, 1.9)
Hypertension	-5.0	-0.1	2.9	2.3
	(-8.4–1.7)	(-4.6, 2.8)	(-1.3, 7.2)	(-2.8, 7.5)
**Difference in outcome associated with a 10% difference in attendance**				
Outcome	Black Female	Black Male	White Female	White Male
Systolic BP(mm/Hg)	-1.9	-1.1	0.2	1.1
	(-3.6, -0.3)	(-3.1, 0.9)	(-0.8, 1.3)	(-0.1, 2.3)
Diastolic BP(mm/Hg)	-1.0	-0.4	0.6	0.3
	(-1.9, -0.1)	(-1.6, 0.0)	(-0.1, 1.1)	(-0.3, 1.0)
Hypertension	-3.7	0.1	1.1	1.3
	(-6.5, -2.0)	(-3.2, 3.3)	(-1.4, 3.6)	(-1.2, 3.9)

^a^ The model includes linear and quadratic terms for age centered at the sample mean, indicator variable for NHANES sample, indicator variables for birth year and indicator variables for state of birth.

^b^ Estimates from models with continuous blood pressure outcomes can be interpreted as difference in mmg/Hg associated with 10% change in school term length. Estimates from models for hypertension can be interpreted as the difference in percentage points in the probability of hypertension associated with 10% change in school term length.

Years of schooling (without corrections for term length) were associated with small differences in SBP, DBP and probability of hypertension for black women, white men and white women, although these differences were not all statistically significant (Table 3). Among black men, uncorrected years of schooling did not statistically significantly predict of SBP, DBP, or hypertension. Among black women, the effect estimates for years of schooling *corrected* for term length were consistently slightly larger than the effect estimates from models using years of schooling without correction for term length (Table 3). For example, among black women, a one-year difference in uncorrected years of schooling was associated with 1.4 percentage point (95% CI = -2.6, -0.3) difference in prevalence of hypertension; in the same model, replacing uncorrected with term-length-corrected years of schooling, a one year increase in schooling predicted a 1.7 percentage point (95% CI = -2.9, -0.6) difference in risk of hypertension. Among white men and white women, the regression coefficients for education were generally similar in the models with versus without corrections for school term length.

**Table 3:** Estimated differences and 95% confidence intervals for systolic and diastolic blood pressure and risk of hypertension associated with years of schooling (uncorrected and corrected for school term length differences), by sex and race, NHANES I & II^a^,^b^.

**Difference in outcome associated with a difference of a year in schooling**				
*Outcome*	Black Females	Black Males	White Females	White Males
*Systolic BP (mmHg)*	-0.7	-0.3	-0.8	-0.2
	(-1.3, 0.2)	(-0.8, 0.3)	(-1.0, -0.6)	(-0.3, 0.0)
*Diastolic BP (mmHg)*	-0.5	-0.2	-0.4	-0.1
	(-0.8, -0.2)	(-0.5, 0.1)	(-0.5, -0.3)	(-0.2, 0.1)
*Hypertension*	-1.4	0.4	-1.5	-0.4
	(-2.6, -0.3)	(-1.5, 0.8)	(-2.0, -1.0)	(-0.9, 0.1)
**Difference in outcome associated with a difference of a year in schooling correcting for STL**				
*Outcome*	Black Females	Black Males	White Females	White Males
*Systolic BP(mmHg)*	-0.9	-0.4	-0.8	-0.1
	(-1.5, -0.3)	(-1.0, 0.3)	(-1.0, -0.6)	(-0.3, 0.0)
*Diastolic BP(mmHg)*	-0.6	-0.2	-0.4	-0.1
	(-0.9, -0.2)	(-0.6, 0.1)	(-0.5, -0.3)	(-0.2, 0.1)
*Hypertension*	-1.7	0.5	-1.5	-0.4
	(-2.9, -0.6)	(-1.8, 0.8)	(-1.9, -1.0)	(-0.9, 0.1)

^a^ The model includes linear and quadratic terms for age centered at the sample mean, indicator variable for NHANES sample, and indicator variables for birth year for state of birth.

^b^ Estimates from models with continuous blood pressure outcomes can be interpreted as difference in mmHg associated with 10% change in school term length. Estimates from models for hypertension can be interpreted as the difference in percentage points in the probability of hypertension associated with 10% change in school term length

Racial differences in blood pressure and hypertension risk were clinically and statistically significant (Table 4). Accounting for years of schooling attenuated the racial disparity in blood pressure and hypertension risk in the models for men and women while improving model fit as measured by the Wald tests. For example, on average, black women had a systolic blood pressure 8.0 mmHg (95% CI = 6.3, 9.7) higher than white women in the model without accounting for years of schooling; this coefficient was attenuated to 7.3 mmHg (95% CI = 5.7, 8.9) when years of schooling was included. The estimated disparity was somewhat further attenuated when accounting for STL corrected years of education: black women had a systolic blood pressure that was 6.8 (95% CI = 5.2, 8.5) higher than white women in the model including the STL corrected measure. To compare the regression coefficients for race between the models with and without the correction for years of schooling, we bootstrapped the difference in the race regression coefficients in the model using the uncorrected years of schooling and the model with the corrected years of schooling 500 times. The racial difference in the models with the uncorrected years of schooling and the corrected years of schooling but bootstrapping the difference was consistently larger than the estimated racial difference in the models with the corrected years of schooling according (Table 5). According to our bootstrapped estimates, the estimated racial disparities in the model for SBP, DBP and hypertension adjusted for uncorrected years of education was statistically significantly larger than the racial disparity estimated in the corresponding models adjusted for the corrected years of education in the female subpopulation. For example, among females, the estimated racial disparities in the model for DBP adjusted for uncorrected years of education was 0.21 mm Hg (95% CI = 0.15, 0.27) larger than the racial disparity estimated in the model for DBP adjusted for STL corrected years of education. Among males, the estimated racial disparities in the models for SBP, DBP and hypertension adjusted for uncorrected years of education was larger than the racial disparity estimated for the corresponding models adjusted for corrected years of education but such differences was only statistically significant for SBP and hypertension. For example, among males, the estimated racial disparities in the model for DBP adjusted for uncorrected years of education was 0.06 mm Hg (95% CI = -0.01, 0.13) larger than the racial disparity estimated in the model for DBP adjusted for STL corrected years of education.

**Table 4:** Racial differences in blood pressure and hypertension prevalence with and without adjustment for years of schooling or years of schooling corrected for school term-length, NHANES I & II^c^.

	Adjusted for sociodemographics	+Uncorrected years of schooling	+Corrected years of schooling	Adjusted for sociodemographics	+Uncorrected years of schooling	+Corrected years of schooling
*Outcome*: *Systolic BP (mmHg)*						
Black	8.0	7.3	6.8	5.6	5.4	5.3
	(6.3, 9.7)	(5.7, 8.9)	(5.2, 8.5)	(3.7, 7.6)	(3.4, 7.4)	(3.2, 7.3)
Education(years)	—	-0.8	-0.8	—	-0.2	-0.2
		(-1.0, -0.6)	(-1.0, -0.6)		(-0.4, 0.0)	(-0.4, 0.0)
*Outcome*: *Diastolic BP (mmHg)*						
Black	5.5	5.2	5.0	4.0	3.8	3.8
	(4.2, 6.7)	(4.0, 6.4)	(3.8, 6.2)	(2.6, 5.4)	(2.5, 5.2)	(2.4, 5.2)
Education (years)		-0.4	-0.4		-0.1	-0.1
		(-0.5, -0.3)	(-0.6, -0.3)		(-0.2, 0.0)	(-0.2, 0.0)
*Outcome*: *Hypertension*						
Black	15.6	14.5	13.8	9.6	9.4	9.2
	(12.1, 19.2)	(10.8, 18.1)	(10.1, 17.5)	(4.8, 14.5)	(4.6, 14.3)	(4.3, 14.0)
Education (years)	—	-1.4	-1.4	—	-0.4	-0.4
		(-1.9, -1.0)	(-1.9, -1.0)		(-0.9, 0.0)	(-0.9, 0.0)

^c^ All models include linear and quadratic terms for age centered at the sample mean, an indicator variable for NHANES sample, indicator variables for birth year and indicator variables for state of birth. Models with uncorrected years of education also included self-reported years of education centered at 12^th^ grade for both females and males. Models with “corrected years of schooling” also included years of education *corrected* for differences in racial school term length and centered at 11^th^ grade for females and 12^th^ grade for males.

**Table 5:** Difference in race regression coefficient in the models using uncorrected years of schooling compared to the models with the corrected years of schooling, by outcome and gender subpopulation^d^.

	Females	Males
*Outcome*: *Systolic BP (mmHg)*	0.44 (0.34, 0.54)	0.11 (0.00, 0.21)
*Outcome*: *Diastolic BP (mmHg)*	0.21 (0.15, 0.27)	0.06 (-0.01, 0.13)
*Outcome*: *Hypertension*	0.006 (0.005, 0.009)	0.003 (0.000, 0.005)

^d^ Bootstrapped differences and 95% CI for the race regression coefficients in the model using the uncorrected years of schooling and the model with the corrected years of schooling.

## Discussion

Much of previous research on education and health using years of schooling has not accounted for differences in school term length across time, geography and race. Although education researchers have long recognized the implicit assumption that years of schooling are equivalent across multiple dimensions is unlikely to be true [11,19], ours is among the first studies to attempt to correct for such differences when considering a health outcome, using a nationally representative sample. We found that longer state average school term length, a measure of school quality, was associated with lower blood pressure and prevalence of hypertension in adulthood for black women. Additionally, correcting years of schooling for historical differences in term length slightly attenuated the effects estimates associated with race. Using naive measures of education that do not account for quality differences may understate the role of schooling in racial disparities.

An emerging body of research has shown educational quality is associated with health gains. [34–36] Our findings are consistent with a recent study which showed improvements in school quality brought about by school desegregation were associated with better self-rated health in adulthood among blacks.[37] Recent work from Frisvold and Golberstein [38] also found school term length to be associated with racial differences in disability and self-rated health. As conditions such as hypertension are often conceptualized as distal precursors to disability in common disability frameworks [39], our results for hypertension specifically are in line with their findings.

While the consistent, statistically significant effect of school term length on blood pressure among black females supports a resource substitution effect, where education’s beneficial effect on health is greater for people wit h fewer alternative resources [24,25,40], the lack of an effect for white females and black males suggest a more complex story. We hypothesize heterogeneous effects of education on hypertension may reflect gender and race-specific opportunities available for converting education to other socioeconomic gains. Changes in school term length and other aspects of education in isolation may not be enough to ensure health benefits. Black women in our study may have been better positioned to use increases in human capital (i.e. education) to obtain better health, because of the socioeconomic employment policies enacted during this period in the US. Equal employment opportunity (EEO) legislation and Civil Rights policies passed in the 1960s and 1970s lead to improved economic positions of blacks as measured by ratios of black to white earnings or ratios of measures of occupational position.[41] These new employment opportunities accrued disproportionately to black women rather than black men, in particular via public sector jobs [42,43]. By primarily benefitting black females, these employment policies may have allowed black women to more effectively utilize any educational advantage to achieve health improvements.

We hypothesize that the opportunities of resource transformation need to be in place for disadvantaged groups to use changes in school quality or quantity to achieve better health. Structural barriers, such as entrenched racial or sex discrimination or other macro-level contextual conditions (e.g. economic recessions), prevent some individuals from transforming education into resources such as increased income and higher occupational or social status. On the other hand, individuals who have other socio-economic advantages may be able to access these downstream resources *regardless of educational quality*. For these people, school quality may likewise have a weak relationship with health. Further research must expand upon these initial results and elucidate what aspect of longer school years improves health, which domains of health are influenced, which subpopulations are most affected and the processes that need to be in place for health improvements to occur.

Previous research has outlined and explored pathways and mechanisms by which education may impact health and health-related behaviors including socioeconomic indicators (e.g. employment status, occupation, income, social status and cognitive skills (e.g. acquisition of specific health knowledge, through improved information processing and decision-making skills). [11, 44–47] Our results support such findings. The resources gained through longer time spent in school and, by extension, classroom time may include factual and content knowledge of the curriculum, as well as a range of other fluid cognitive skills or non-cognitive skills such as behavior, motivation and self-control.[48] Further research is need to determine the various mechanisms linking school term length to adult hypertension risk as well as whether these mechanisms according to subpopulations and other.

Our results are consistent with a life-course perspective suggesting that historical differences in social policies affecting young children contribute to persistent racial disparities in adult hypertension. The process leading to cardiovascular diseases starts early in life and is influenced over the life-course by a multitude of modifiable risk factors including smoking, diet, and healthcare access. Education directly influences a host of behavioral and psychosocial processes throughout the life-course that determine blood pressure and risk for hypertension in adulthood. Recent studies have started to stress the role of environmental and biological primordial risk factors for adult cardiovascular health.[49,50] Our study extends previous research on primordial prevention of cardiovascular disease by stressing the role of educational policy and school quality. Longer school years, and other investments in the determinants of primordial risk factors, may provide a means to improve population health and reduce cardiovascular health disparities.[51]

This study has several limitations. State average school term length is a very imperfect proxy for school quality experienced by individual children. Other institutional features not available in our dataset, such as teacher training and expenditures, are also likely to affect school quality. There may also be misclassification because we assume that individuals went to school in their state-of-birth. A fraction of children probably moved to other states before attending elementary school. While we are unable asses measurement error associated with using state of birth as the state in which respondent began schooling in our sample, we did find that approximately 91% of children between the ages of 5–12 in the 1940 Census IPUMS data (birth cohorts also in our sample) resided in the state they were born in, suggesting limited migration between state of birth and state beginning elementary school. Within-state heterogeneity in school term length would probably be similar to between-state heterogeneity, so the differences we see between corrected and uncorrected educational measures would likely be larger if we had more precise measures of term length at the district level. Another plausible limitation is residual confounding by state-level characteristics associated with school term length. We minimized this potential limitation by including indicator variables for state and year of birth. In order to account for our findings in models adjusted for state and year of birth, potential confounders would need to both affect blood pressure later in life and vary within states and over birth cohorts in a way that was correlated with the changes in school term length. In the models examining racial disparity in the association between years of schooling and hypertension, we would like to acknowledge the association between education and hypertension is likely to be mediated and confounded by various health conditions and behaviors throughout the lifecourse including diet, physical activity and obesity.

Despite these limitations, our study results yield important insights. Our results indicate the experience of a year of schooling for white students differed dramatically from a year for black students in the US. Furthermore, school term length was associated with decreases in blood pressure and hypertension risk among Black females. The associations we present highlight the long term influence of educational policy on economic and health outcomes in later life. Schools may be effective policy instruments to address health disparities [51], especially if coupled with other contextual changes that affect subsequent life stages.
